# Continuous glucose monitor overestimates glycemia, with the magnitude of bias varying by postprandial test and individual – a randomized crossover trial

**DOI:** 10.1016/j.ajcnut.2025.02.024

**Published:** 2025-02-26

**Authors:** Katie M Hutchins, James A Betts, Dylan Thompson, Aaron Hengist, Javier T Gonzalez

**Affiliations:** 1 Centre for Nutrition, Exercise, and Metabolism, University of Bath, Bath, United Kingdom; 2 Department for Health, University of Bath, Bath, United Kingdom; 3 National Institute of Diabetes and Digestive and Kidney Diseases, NIH, Bethesda, MD, United States

**Keywords:** glycemic index, glycemic response, fruit smoothie, metabolism, carbohydrate, capillary, interstitial

## Abstract

**Background:**

Continuous glucose monitors (CGM) are used to characterize postprandial glycemia, yet no study has directly tested how different test foods/beverages alter CGM accuracy.

**Objectives:**

Assess glycemic responses to test foods/drinks using CGM compared with capillary sampling (criterion).

**Methods:**

Fifteen healthy females (*n* = 9) and males (*n* = 6) completed 7 laboratory visits in a randomized crossover design with ≥48 h washout between visits. During each visit, participants consumed an oral carbohydrate challenge comprising either 50 g glucose or equivalent 50 g carbohydrate as whole fruits, 50 g carbohydrate as blended fruit, 50 g carbohydrate as commercially available fruit smoothie, 50 g carbohydrate as commercially available fruit smoothie ingested over 30 ± 4 min, 50 g carbohydrate as commercially available fruit smoothie with 5 g inulin, 30 g carbohydrate as commercially available fruit smoothie. The glycemia was recorded from both CGM and capillary samples every 15 min for 120 min and expressed as incremental areas under the curve. The glycemic index (GI) was calculated relative to 50 g glucose where appropriate. Exploratory analyses examined *1*) interindividual heterogeneity of CGM bias compared with criterion and *2*) whether CGM bias could be improved with adjustment for baseline differences.

**Results:**

CGM-estimated fasting and postprandial glucose concentrations were (mean ± standard deviation) 0.9 ± 0.6 and 0.9 ± 0.5 mmol/L higher than capillary estimates, respectively(both, *P* < 0.001). CGM bias varied by postprandial test such that GI for 50 g carbohydrate as commercially available fruit smoothie was higher with CGM (69; 95% confidence interval: 48, 99) compared with capillary (53; 95% confidence interval: 40, 69; *P* = 0.05). Furthermore, differences in CGM compared with capillary fasting glucose concentrations varied by participant (*P* = 0.001). Unadjusted, CGM overestimated time >7.8 mmol/L by ∼4-fold, and adjustment for baseline differences reduced this overestimate to ∼2-fold (both *P* < 0.01).

**Conclusions:**

CGM overestimated glycemic responses in numerous contexts. At times, this can mischaracterize the GI. In addition, there is interindividual heterogeneity in the accuracy of CGM in estimating fasting glucose concentrations. Correction for this difference reduces, but does not eliminate, postprandial overestimate of glycemia by CGM. Caution should be applied when inferring absolute or relative glycemic responses to foods using CGM, and capillary sampling should be prioritized for accurate quantification of glycemic response.

This trial was registered at clinicaltrials.gov as NCT06333184.

## Introduction

Glycemic responses to foods are an important component of health and can also be used to characterize some properties of foods and other behaviors (e.g., physical activity [1]). Mild sustained increases in glycemia (7.8 mmol/L compared with 5.2 mmol/L) induced by infusion raise fasting glycemia and whole-body insulin resistance and impair β-cell function in healthy people within days [2], and even more modest increases in glycemia induce hepatic insulin resistance [3]. Therefore, characterizing glycemia, even within the normoglycemic range, is of relevance to cardiometabolic health.

The glycemic index (GI) is a way to classify foods based on how much and for how long they increase glycemia after a meal, expressed relative to a reference (typically 100% glucose). The glycemic response to foods can be influenced by the food’s physical structure and the type of carbohydrate and noncarbohydrate (e.g., fiber) components [4,5]. It is recommended that glycemic response to foods be characterized using fingertip capillary or arterialized-venous blood since mixed venous blood can introduce greater variation and reduce sensitivity [5,6]. Capillary blood is, in fact, the criterion method for GI measurement [5].

Continuous glucose monitors (CGM) are increasingly being used to assess glucose responses to foods in people without diabetes [7,8] and were recently approved for over-the-counter purchase in the United States for this purpose [9]. The validity of CGM has largely been based on comparisons against venous or capillary blood glucose measures taken at various times across a day [10]. Whether CGM can effectively reflect glycemic responses and characterize the GI of foods and beverages is currently unclear [11]. Since CGM samples interstitial fluid and indirectly estimates blood glucose concentrations, there is good reason to expect differences between CGM and capillary glucose measures. Since interstitial and capillary blood glucose concentrations are likely to be influenced by glucose fluxes and blood flow (among other factors), different CGM errors could vary by people and with different foods/beverages. Indeed, when different types of CGM are worn simultaneously, glycemic responses to various foods are differentially ranked [12]. If this is systematically different from the ingestion of pure glucose, then this also has implications for the quantification of GI. To date, no study has directly compared the measurement of glycemic responses with CGM compared with criterion methods across several postprandial tests. Accordingly, this study aimed to compare glucose responses to different oral carbohydrate challenges (foods and fluids) when measured using CGM compared with the criterion method of capillary blood sampling. It was hypothesized that due to the lack of direct sampling from blood, CGM would provide different estimates of blood glucose concentrations than the criterion method and that this difference may vary by postprandial test and potential by individuals.

## Methods

### Study design

The study was an open-label, randomized crossover design with 7 experimental conditions separated by at least a 2-d washout between consecutive visits (maximum 3 mo; median 3 d). The protocol received a favorable opinion from the University of Bath, Biomedical Sciences Research Ethics Committee (reference: 3085-4215) and was preregistered at clinicaltrials.gov (NCT06333184). Participants provided informed, written consent prior to participation. The study was conducted in line with the latest version of the Declaration of Helsinki [13]. The current study was performed in line with all of the recommendations for GI testing that are provided by Brouns et al. [5]. Other than the replication of the reference condition. This decision was made based on the trade-offs between the precision of GI estimates and the increase in the number of different postprandial tests available to compare CGM with capillary sampling.

### Participants

Participants were healthy adult males and females from the area local to Bath, United Kingdom (recruited and tested between March 2024 and August 2024) and were aged 18–65 y with a BMI (in kg/m^2^) between 18 and 30. Exclusion criteria included: diagnosis of any form of diabetes; intolerances or allergies to any of the study procedures (e.g., fructose/inulin intolerance); fructose malabsorption; inborn errors of fructose metabolism (e.g., fructokinase deficiency, aldolase B deficiency, fructose-1,6-bisphosphatase deficiency); pregnant or lactating; any condition that could introduce bias to the study (e.g., diagnoses of lipid disorders, including cardiovascular disease, or therapies that alter lipid or glucose metabolism, such as statins or niacin). All exclusion criteria were based on self-reporting. Participants were provided with £210 upon completion of the study.

### Randomization

Following screening (eligibility assessment), participants were allocated the randomly generated sequence of conditions. The condition assigned to the first visit was counterbalanced, and conditions assigned to the remaining visits were performed in a random order (randomly assigned by JTG using www.randomizer.org; Supplemental Table 1).

### Pretrial standardization

In line with GI testing guidelines [5], participants avoided unusual vigorous activity (i.e., vigorous activity was permitted if this was part of a usual schedule) and ate their normal diet on the day before laboratory visits. The evening meal before visits was a meal of their choice prior to their first trial (no alcohol permitted), which was replicated for subsequent visits (timing and type of meal). Smoking was not permitted on the day of laboratory visits. Compliance with these measures was by self-report. Participants were fitted with a CGM by a member of the research team at a place of convenience ≥24 h prior to the first test drink to avoid reduced accuracy in the initial 24-h wear time [10]. These monitors remained in place for ≤14 d or were replaced if they failed or became loose.

### Laboratory visits

On laboratory visit days, participants arrived in a rested, overnight fasted state. Body mass was recorded. Participants were seated for the first blood sample and for the remainder of the test period, with only brief short walks to the toilets permitted. Mean (range) ambient temperature, humidity, and pressure were 20.2°C (18.4–22.3°C), 33.4% (30.0–42.0%), and 764 mmHg (736–791 mmHg). A baseline fingerstick blood sample was taken in duplicate. Participants then began ingestion of the test drink/food, and further blood samples were taken in duplicate at 15 min, 30 min, 45 min, 60 min, 90 min, and 120 min after the first sip/mouthful. The nutrient composition of the test drinks/food is displayed in Table 1.

**TABLE 1:** Main laboratory visits and drink/food composition.

Condition	Carbohydrate (g)	Volume (mL)	Ingestion time (min)	Fat (g)	Protein (g)	Fiber (g)	Ingredients/description	Fructose: glucose (ratio)	Hypothesized response relative to PRODUCT
CONTROL^1^	50	417	<5 min	-	-	-	50 g glucose (55 g dextrose powder accounting for hydration) plus 417 mL water	0	↑
PRODUCT^2^	50	417	<5 min	<0.5	<0.5	5	417 mL of commercially available “mangoes, passion fruits, and apples” fruit smoothie providing 50 g carbohydrate	1.8	N/A
WHOLE^7^	50	417	<5 min	<0.5	<0.5	5	Apples (51%), mango (16%), banana (16%), orange (12%), passionfruit (3%, peach (2%), lime (0.4%, recipe matched to PRODUCT) eaten as whole fruit with added water as needed to match the volume	1.2	↓
BLEND^6^	50	417	<5 min	<0.5	<0.5	5	Apples (51%), mango (16%), banana (16%), orange (12%), passionfruit (3%, peach (2%), lime (0.4%, recipe matched to PRODUCT) eaten as blended fruit with added water as needed to match the volume	1.2	=
SLOW^4^	50	417	>25 min, <35 min	<0.5	<0.5	5	417 mL of commercially available “mangoes, passion fruits, and apples” fruit smoothie providing 50 g carbohydrate ingested slowly over 25–35 min	1.8	↓
FIBER^3^	50	417	<5 min	<0.5	<0.5	10	417 mL of commercially available “mangoes, passion fruits, and apples” fruit smoothie providing 50 g carbohydrate with 5 g of added inulin	1.8	↓
DOSE^5^	30	250	<5 min	<0.5	<0.5	4	250 mL of commercially available “mangoes, passion fruits, and apples” fruit smoothie providing 30 g carbohydrate	1.8	↓

Up arrow = increase, down arrow = decrease.

N/A, Not applicable.

1 50 g glucose,

2 commercially available fruit smoothie ingested alone within 5 min,

3 with 5 g inulin,

4 ingested slowly over ∼30 min,

5 ingested as a 30 g dose of carbohydrate,

6 ingested as blended fruit with the same ingredients, or

7 ingested as whole fruit, *n* = 15 healthy adults.

### Measurements and analyses

Capillary blood glucose concentrations were measured in duplicate at each timepoint using a handheld monitor (Abbott Optium Neo Blood Glucose Monitor), and interstitial glucose concentrations were determined using a CGM (Abbot Freestyle Libre 2). Two to three sensors were used for each participant, depending on the schedule of visits and sensor failure. Capillary sampling was performed with minimal squeezing of the fingertip, and heating was not needed. The handheld glucose monitor was calibrated prior to each test according to the manufacturer’s instructions (Glucose control solution; MediSense). Lower and upper limits of detection for this device are 1.1 mmol/L and 27.7 mmol/L, respectively. Data all fell within these ranges. The CGM was factory-calibrated by the manufacturer.

### Sample size justification

When adhering to best practice guidelines for GI methodology [5], 10 participants provide a reasonable degree of power (>80%) and precision for most purposes of measuring GI, and differences that require larger sample sizes are unlikely to be biologically meaningful for people without diabetes [5]. We therefore recruited 15 participants to account for a potential dropout of ≤33%. Further justification for this was based on prior evidence that suggests the GI of the 50 g carbohydrate as a commercially available fruit smoothie (PRODUCT) is <40 (i.e., low [14]). With test foods that have a GI of 40, 15 participants should provide a margin of error for a 95% confidence interval (CI) of <10 GI and provide >80% power to detect a difference in GI of ≥12 [5], and thus is sufficient to distinguish between most GI categories.

### Statistical analyses

Data were analyzed in Prism v10.2.3 (GraphPad Software). The mean of duplicate capillary samples was calculated for the criterion measure. The coefficient of variation (percentage) for duplicate samples was also calculated to establish the between-sample variance. The distribution of data was assessed by visual inspection of Q-Q plots in addition to the Shapiro-Wilk test. Glucose concentrations (capillary and interstitial) were converted into the incremental AUC (iAUC) using the trapezoidal rule using timepoints 0 min, 15 min, 30 min, 45 min, 60 min, 90 min, and 120 min [15]. The iAUC for PRODUCT, 50 g carbohydrate as whole fruit (WHOLE), 50 g carbohydrate as blended fruit (BLEND), and 50 g carbohydrate as commercially available fruit smoothie with added inulin (FIBER) was expressed as a percentage of 50 g glucose (CONTROL) (i.e., the GI [5]) and classified as either low GI (<56), moderate GI (56–69) or high GI (>69). Since 30 g carbohydrate as a commercially available fruit smoothie (DOSE) and 50 g carbohydrate as a commercially available fruit smoothie ingested over ∼30 min (SLOW) did not adhere to GI testing guidelines (i.e., consumption within 5–10 min for fluids, 10–20 min for solids, and 50 g available carbohydrate), these conditions were assessed with iAUC but not with GI.

Paired Student’s *t*-tests (or Wilcoxon matched-pairs signed rank tests where appropriate; lower case *p* denotes use of *t*-test and upper case *P* denotes use of Wilcoxon test) were performed to assess the following prespecified comparisons: capillary compared with interstitial GI for PRODUCT, WHOLE, BLEND, and FIBER, and capillary compared with interstitial iAUC for all conditions. One-way analysis of variance (ANOVA) was used to compare *1*) the iAUC between each condition using capillary sampling and *2*) the iAUC between each condition using interstitial sampling. Two-way (sampling method and condition), repeated-measures ANOVA (with Greenhouse-Geisser correction) were used to assess the sampling method-by-condition interaction for the iAUC, peak glucose concentrations, and time-to-peak glucose concentrations. Two-way (sampling method by time) repeated-measures ANOVA was used to assess the sampling method by time interaction for each condition. To examine the potential impact on interpretations of time-in-range (or time-out-of-range), the time spent above the American Diabetes Association secondary target range (7.8 mmol/L [16]) was calculated for each participant and summed across all 7 laboratory visits. This value was also chosen on the basis of the magnitude of glycemia shown to elicit increases in fasting glycemia and insulin resistance in normoglycemic individuals [2]. To assess the potential for interindividual heterogeneity of the accuracy of interstitial sampling to estimate capillary glucose concentrations, the 7 replicates of fasting glucose concentration for each participant were used, and differences between participants were assessed by a 1-way ANOVA. Pearson’s product-moment correlation coefficients were used to quantify the associations between participant characteristics and the interstitial-capillary difference in fasting glucose concentrations. Thresholds of 0.1, 0.3, and 0.5 were used to infer correlations as small, moderate, and large, respectively [17]. All data are presented as mean ± SD (or 95% CI) unless stated otherwise, and the α level was set at 0.05. Since all GI data displayed evidence of non-normal distribution of raw values and of residuals (which is expected due to the nature of GI being a ratio), the GI values are presented as geometric means, and comparisons between CGM and the criterion method were assessed by Wilcoxon matched-pairs signed rank test. The minimum clinically-important differences (MCID) were determined using prior literature [18]. For GI, the MCID was 10 on the basis that this reflects the boundary between low and high GI, and the MCID for fasting glucose concentration was considered to be 0.5 mmol/L on the basis that this is the smallest difference shown to be associated with mortality [19]. An exploratory analysis was also performed to establish whether the CGM method could be improved by a correction factor. The difference in CGM compared with criterion glucose concentration in the fasted state was used to adjust the CGM values on an individual basis (e.g., if CGM = 5 mmol/L in the fasted state, and criterion = 4.5 mmol/L, all CGM data for that participant during that postprandial test were expressed as the reported CGM value minus 0.5 mmol/L). Where this adjustment has been performed, this is expressed as CGM_adj_. This was not performed for GI data, iAUC, or time-to-peak, as the adjusted baseline bias makes no difference to these data.

## Results

Fifteen participants were recruited (9 females and 6 males), and all participants completed all laboratory visits. The mean age ± SD, stature, body mass, and BMI of participants were 34 ± 14 y, 1.75 ± 0.10 m, 74 ± 10 kg and 24.05 ± 2.6. The coefficient of variation for duplicates of fasting and postprandial capillary glucose concentrations were 3.5 ± 2.8% and 3.2 ± 2.8%, respectively, and neither were significantly different from the 3% recommended as threshold for use (*P* > 0.05 for all timepoints). The mean start time of tests was 08:51 (range 06:50–11:00) and was standardized within individuals to within 1 h between tests. The time taken to ingest CONTROL was 3 ± 2 min, PRODUCT was 2 ± 1 min, FIBER was 3 ± 2 min, DOSE was 2 ± 1 min, BLEND was 4 ± 1, WHOLE was 7 ± 3, and SLOW was 30 ± 4 min.

**FIGURE 1:**
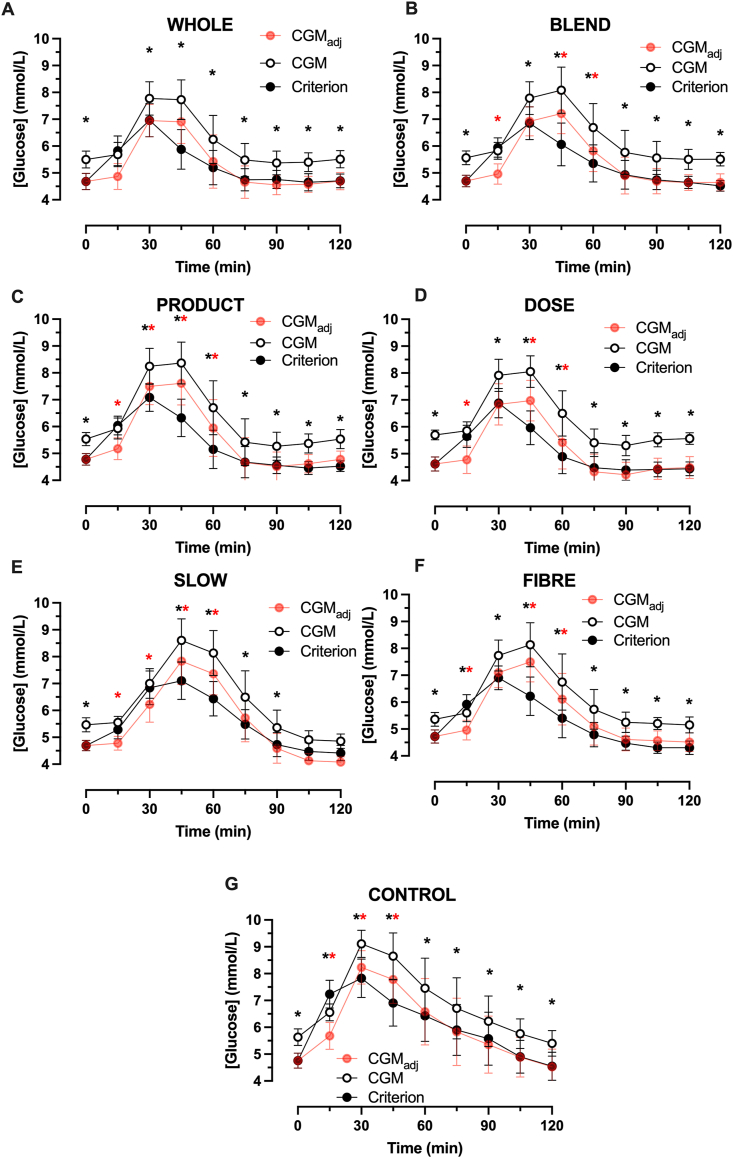
Glucose concentrations measured using continuous glucose monitor (CGM) or capillary blood (criterion) in response to ingestion of 50 g of carbohydrate as whole fruit (WHOLE; A), 50 g carbohydrate as blended fruit (BLEND; B), 50 g carbohydrate as commercially available fruit smoothie ingested within 5 min (PRODUCT; C), as a 30 g carbohydrate as commercially available fruit smoothie (DOSE; D), 50 g carbohydrate as commercially available fruit smoothie ingested over ∼30 min (SLOW; E), 50 g carbohydrate as commercially available fruit smoothie ingested with 5 g inulin (FIBER; F), and 50 g glucose (CONTROL; G), *n* = 15 healthy adults. Data are mean ± 95% CI. The black asterisk relates to *P* < 0.05 for CGM compared with the criterion. The red asterisk relates to *P* < 0.05 for CGM_adj_ compared with the criterion. CGM_adj_ represents the CGM values adjusted for the difference in fasting glucose concentrations compared with the criterion method. CGM_adj_, continuous glucose monitor adjusted for the difference in fasting glucose concentrations relative to capillary samples; CI, confidence interval.

Glucose concentrations over time for each condition are presented in Figure 1. The main effects of time, main effects of the sampling method, and time-by-sampling method interaction effects were detected in all conditions (all *P* < 0.001). The typical pattern across all conditions was that, in the fasted state, CGM reported higher glucose concentrations than the criterion. Following the ingestion of the test drinks/foods, glucose concentrations rose to a greater extent with CGM than with the criterion method. This overestimation persisted, albeit to a lesser extent, with CGM_adj_.

**FIGURE 2:**
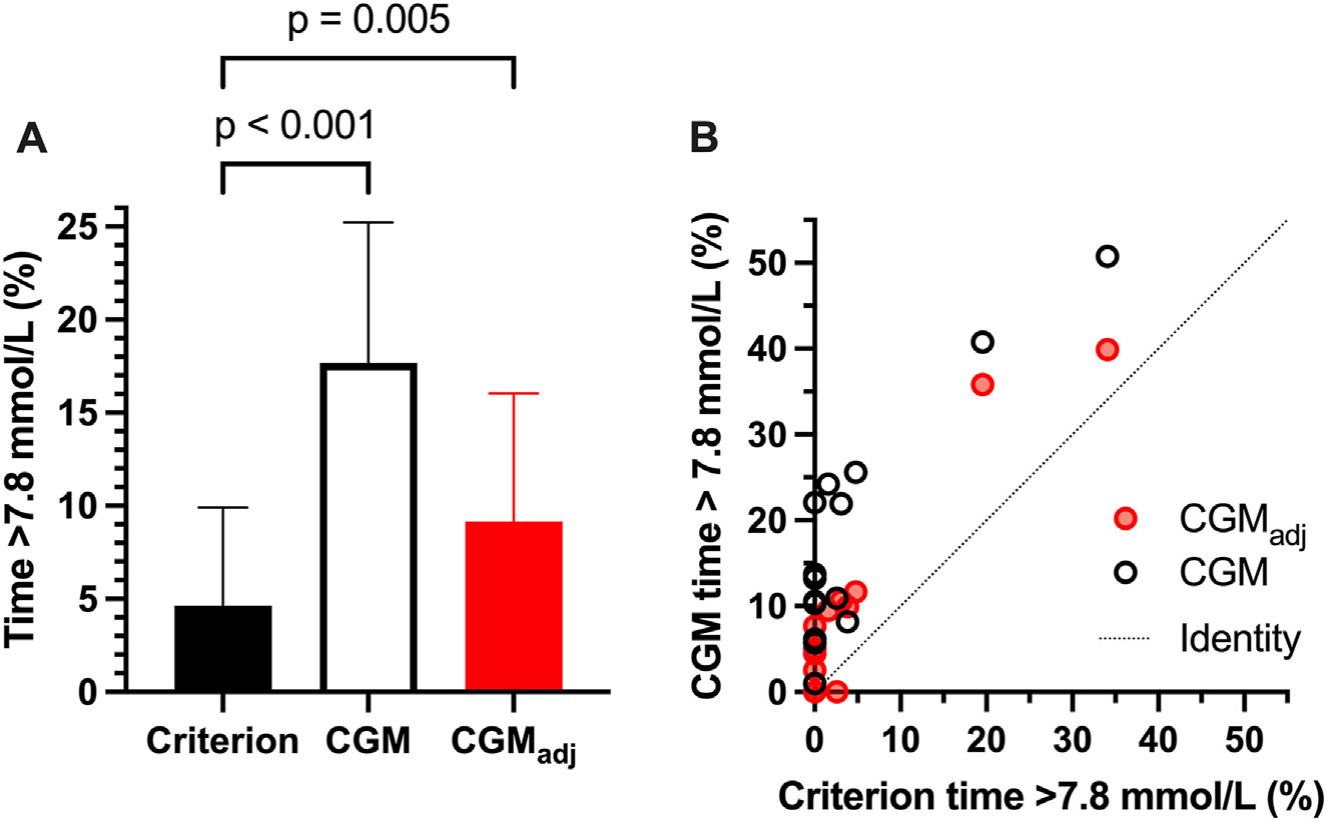
Time out of range (>7.8 mmol/L) when the glucose concentrations measured using continuous glucose monitor (CGM), CGM adjusted for the difference in fasting glucose from the criterion method (CGM_adj_) or capillary blood (criterion) across postprandial periods were summed expressed as means ± 95% CI (A) and as the correlation between criterion and CGM (B), *n* = 15 healthy adults. CI, confidence interval.

The increase in glucose concentrations measured with CGM compared with the criterion method resulted in a >3.8-fold increase in the time spent above 7.8 mmol/L across the 7 conditions (Figure 2A) and was still overestimated by ∼2-fold with CGM_adj_. CGM overestimated time >7.8 mmol/L in all participants, with slight improvement when expressed as CGM_adj_ (Figure 2B).

**FIGURE 3:**
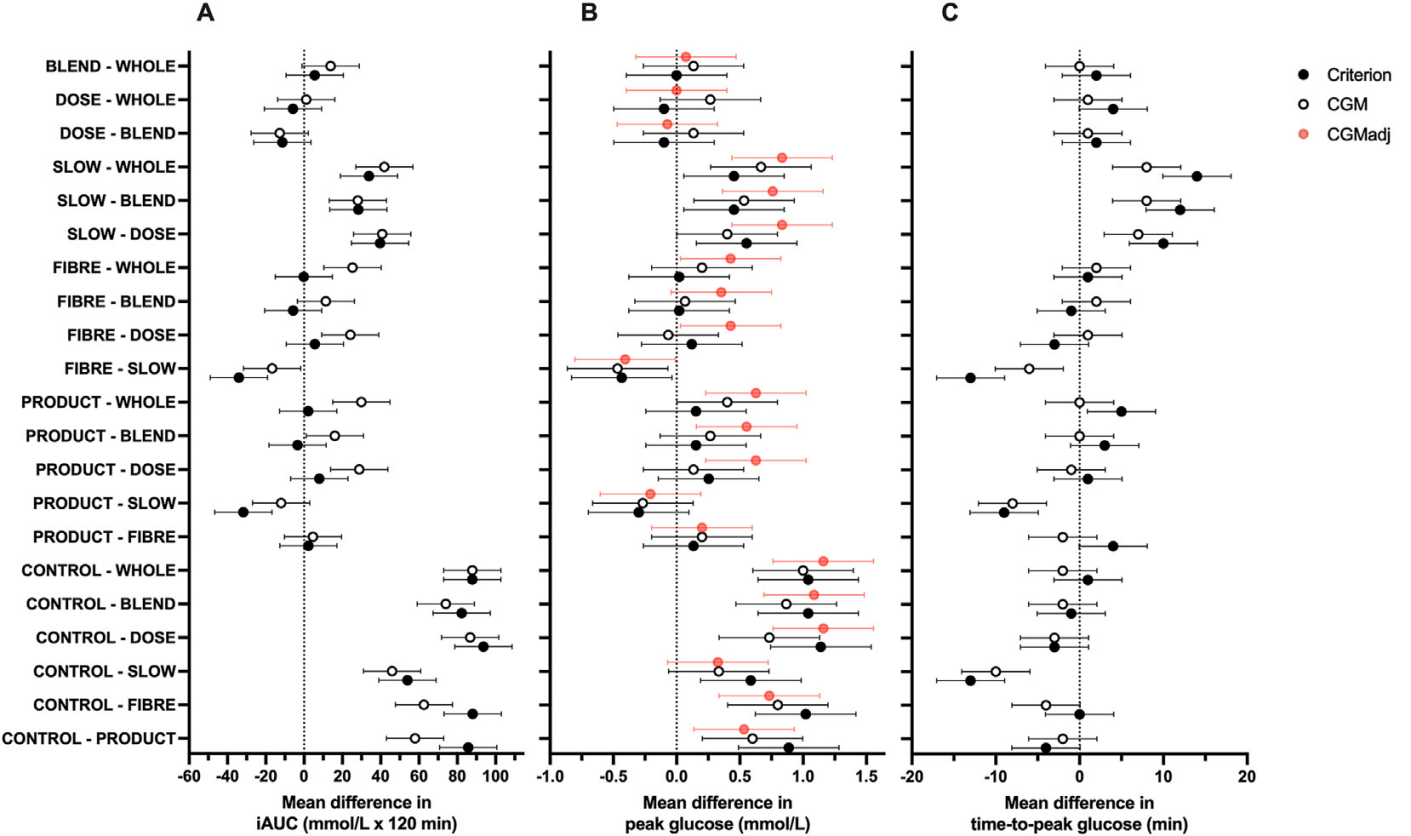
Mean differences (95% CI) in the incremental AUC(iAUC; A), in peak (B) and time-to-peak (C) glucose concentrations using continuous glucose monitor (CGM) CGM adjusted for the difference in fasting glucose from the criterion method (CGM_adj_) or capillary blood (criterion) in response to ingestion of 50 g of carbohydrate as whole fruit (WHOLE), 50 g carbohydrate as blended fruit (BLEND), 50 g carbohydrate as commercially available fruit smoothie ingested within 5 min (PRODUCT), as a 30 g carbohydrate as commercially available fruit smoothie (DOSE), 50 g carbohydrate as commercially available fruit smoothie ingested over ∼30 min (SLOW), 50 g carbohydrate as commercially available fruit smoothie ingested with 5 g inulin (FIBER), and 50 g glucose (CONTROL), *n* = 15 healthy adults. CI, confidence interval; iAUC, incremental area under the glucose curve.

The glucose iAUC was consistently higher for CGM than the criterion (main effect of sampling method, *P* = 0.009), and the glucose iAUC differed across conditions (main effect of condition, *P* < 0.0001). No sampling method-by-condition interaction effect was detected for glucose iAUC (*P* = 0.194). *Post hoc* differences in glucose iAUC between conditions are displayed in Table 2 and Figure 3A. The rank order for iAUC (from highest to lowest) based on the criterion method was CONTROL > SLOW > BLEND > PRODUCT > WHOLE > FIBER > DOSE. Whereas the rank order for iAUC (from highest to lowest) based on the CGM method was CONTROL > SLOW > PRODUCT > FIBER > BLEND > DOSE > WHOLE. Peak glucose concentrations were higher with CGM compared with the criterion method (main effect of sampling method, *P* < 0.0001), and peak glucose concentrations differed across conditions (main effect of condition, *P* < 0.001). No sampling method-by-condition interaction effect was detected for peak glucose concentrations (*P* = 0.903). *Post hoc* differences in peak glucose between conditions are displayed in Table 2 and Figure 3B. Time-to-peak glucose was around 5–12 min later for CGM compared with the criterion method (main effect of sampling method, *P* < 0.0001), and time-to-peak glucose concentrations differed across conditions (main effect of condition, *P* < 0.001). No sampling method-by-condition interaction effect was detected for time-to-peak glucose concentrations (*P* = 0.481). *Post hoc* differences in time-to-peak glucose concentrations between conditions are displayed in Table 2 and Figure 3C.

**TABLE 2:** Incremental AUC, peak concentrations, and time-to-peak concentrations for glucose measured using continuous glucose monitor or capillary blood following ingestion of various test drinks/foods in healthy adults.

Condition	iAUC (mmol/L x 120 min)				Peak (mmol/L)				Time-to-peak (min)			
	Criterion^1^	CGM^2^	Mean difference (95% CI)	*P* value	Criterion	CGM	Mean difference (95% CI)	*P* value	Criterion	CGM	Mean difference (95% CI)	*P* value
CONTROL^3^	181 ± 114	181 ± 119	1 (–17, 18)	0.935	8.1 ± 1.4	9.5 ± 1.4	1.4 (0.8, 2.0)	0.001	29 ± 19	37 ± 11	8 (1, 15)	0.041
PRODUCT^4^	95 ± 56	123 ± 76	29 (9, 48)	0.012	7.2 ± 0.9	8.8 ± 1.2	1.6 (1.2, 2.0)	<0.001	33 ± 8	39 ± 9	6 (2, 10)	0.009
FIBER^5^	93 ± 50	119 ± 54	26 (15, 38)	0.001	7.1 ± 0.9	8.5 ± 1.4	1.4 (1.0, 1.9)	<0.001	29 ± 9	41 ± 9	12 (9, 15)	<0.001
SLOW^6^	127 ± 57	135 ± 66	9 (–9, 27)	0.358	7.5 ± 1.0	9.1 ± 1.3	1.5 (1.0, 2.1)	<0.001	42 ± 10	47 ± 13	5 (–3, 13)	0.238
DOSE^7^	87 ± 61	94 ± 59	8 (–13, 28)	0.488	7.0 ± 1.0	8.5 ± 0.8	1.6 (1.2, 1.9)	<0.001	32 ± 10	40 ± 9	8 (4, 12)	0.001
BLEND^8^	98 ± 77	107 ± 68	9 (–8, 26)	0.307	7.1 ± 1.0	8.4 ± 1.4	1.3 (1.0, 1.7)	<0.001	30 ± 8	39 ± 8	9 (5, 13)	<0.001
WHOLE^9^	93 ± 68	93 ± 48	1 (–22, 24)	0.947	7.1 ± 1.1	8.3 ± 1.1	1.2 (0.8, 1.6)	<0.001	28 ± 8	39 ± 8	11 (8, 14)	<0.001

Data are mean ± SD (or 95% CI).

Abbreviations: AUC, area under the curve; CGM, continuous glucose monitor; CI, confidence interval; iAUC, incremental area under the curve; SD, standard deviation.

1 Capillary blood,

2 continuous glucose monitor,

3 50 g glucose,

4 commercially available fruit smoothie ingested alone within 5 min,

5 with 5 g inulin,

6 ingested slowly over ∼30 min,

7 ingested as a 30 g dose of carbohydrate,

8 ingested as blended fruit with the same ingredients, or

9 ingested as whole fruit. *n* = 15 healthy adults.

When using the criterion method, the GI for WHOLE, BLEND, FIBER, and PRODUCT were all “low” (all *P* > 0.05 for comparisons between test drinks/food; Supplemental Figure 1). In contrast, when using CGM, the GIs for WHOLE, BLEND, and PRODUCT were medium, and the GI for FIBER was high (*P* = 0.05 for FIBER and PRODUCT when comparing CGM with the criterion method; Supplemental Figure 1).

**FIGURE 4:**
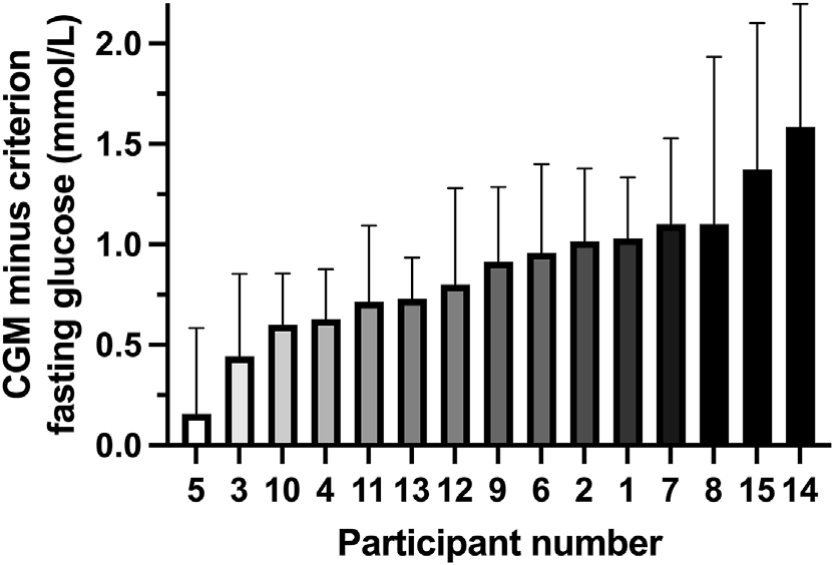
Difference in continuous glucose monitor (CGM) compared with criterion (capillary blood) glucose concentration in the fasted state. Each bar represents the mean ± 95% CI of 7 replicates for each participant, *n* = 15 healthy adults. CI, confidence interval.

**FIGURE 5:**
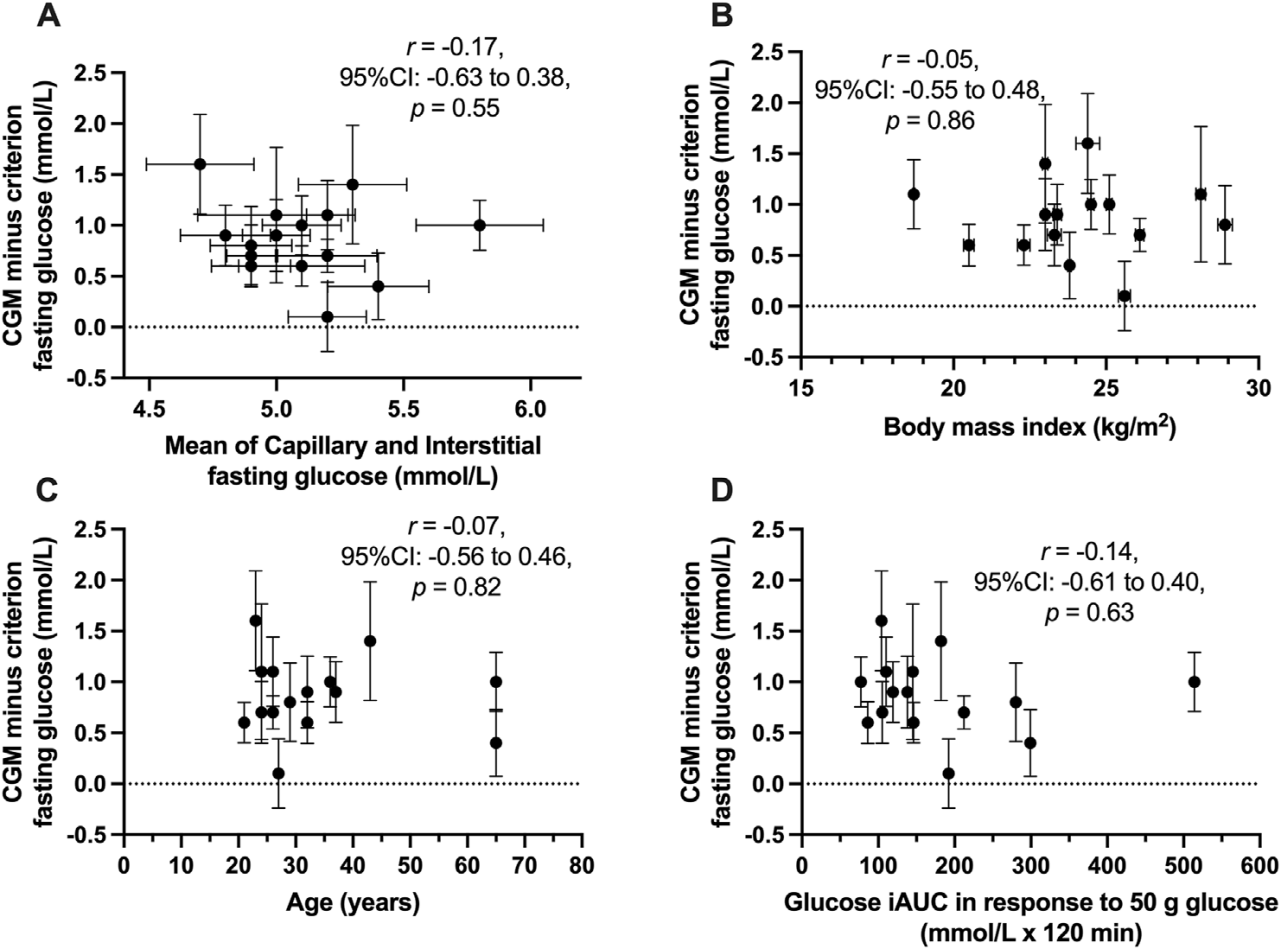
Difference in fasting continuous glucose monitor (CGM) compared with criterion (capillary blood) glucose concentration in relation to the mean of fasting CGM and criterion glucose concentration (A), BMI (B), age (C), and glucose tolerance (D). Each dot represents a single participant; error bars represent the 95% CI of 7 replicates for each participant where available, *n* = 15 healthy adults. BMI, body mass index; CI, confidence interval; iAUC, incremental area under the curve.

The degree to which fasting glucose concentrations were higher with CGM compared with criterion varied by participant (*P* < 0.001; Figure 4). Furthermore, the between-participant SD in this CGM bias was higher than the MCID of 0.5 mmol/L (between-participant SD: 0.6 ± 0.1 mmol/L). The interindividual heterogeneity of CGM compared with criterion difference could not be explained by fasting glucose concentrations (Figure 5A), BMI (Figure 5B), age (Figure 5C), or glucose tolerance (Figure 5D). The correlation between days of sensor use and the CGM compared with criterion difference was also negligible (r = 0.03; 95% CI: –0.17, 0.22; *P* = 0.79; Supplemental Figure 2A). The mean within-device SD was 0.35 ± 0.09 mmol/L, compared with a mean between-device SD of 0.32 ± 0.26 mmol/L (Supplemental Figure 2B).

## Discussion

These data demonstrate that the validity of CGM to estimate glycemia depends on both the test food ingested and the participant – specifically, the glycemic response to some foods (such as commercially available smoothies) is systematically overestimated, and fasting glycemia is overestimated more so in some individuals than others. CGM also overestimates time-out-of-range, and calibration of the continuous glucose monitor based on the difference in fasting glucose concentration attenuates, but does not eliminate this overestimatation. Furthermore, the data demonstrate little difference in the GI when the fruit is consumed in whole form or as various forms of smoothie. These findings have numerous implications for the use of CGM to accurately quantify the GI and glycemic responses. For example, companies now encourage people to use CGM to guide lifestyle choices with a target of maintaining blood glucose in a specific range, and these devices can be bought “over-the-counter” [8,9]. These data suggest that CGM may overestimate the time that healthy people spend outside this range and misrepresent the relative glycemic response of certain beverages, thereby misleading people about nutritional strategies to lower postprandial glycemia.

CGM is increasingly used to quantify glycemic responses to meals in healthy adults [7]. Although prevalence is unclear, there are now several companies offering CGM for the general population [8], albeit cost may be a significant barrier to many and bias this population to higher socioeconomic status groups. CGM measures glucose concentrations in interstitial fluid rather than in the circulation and then estimates the systemic glucose concentration. Since most of the bodily tissues receive arterial blood from the heart, arterial glucose concentrations are often the primary measurement of interest in whole-body glucose exposure and glycemic responses/control [5], and sampling from other compartments can introduce artifacts from local tissue metabolism and/or delays in equilibration of glucose between compartments. However, sampling arterial blood is technically challenging, and fingertip capillary blood can provide an adequate representation of arterial blood glucose concentrations without the same risks or technical challenges. Accordingly, fingertip capillary blood is considered the gold-standard method of measuring glycemic responses to foods [5]. Whether the measurement of interstitial glucose concentrations from CGM can adequately quantify glycemic responses to foods has been unclear. The data from the current study demonstrate that CGM overestimates fasting and postprandial glucose concentrations on average and leads to an overestimation of relative hyperglycemia by >3-fold (reduced to ∼2-fold when adjusting/calibrating for baseline differences against the criterion). Furthermore, the degree to which CGM overestimates glycemia depends on the individual and the test food ingested (which unfortunately means that no single correction factor can be used to remove this bias for all individuals in all contexts – i.e., the estimate is neither valid nor reliable between different assessments). Accordingly, the rank order of glycemic response was different for 5 out of the 7 test beverages/foods with CGM compared with the criterion method. This presents several challenges in relation to the use of CGM for quantifying glycemic responses to foods.

The glycemic response to a food is often characterized relative to a reference of CONTROL (or sometimes 50 g carbohydrate from white bread). The iAUC of a test food relative to the reference food is the GI (i.e., an iAUC, which is 50% of the reference food, means the GI is 50). If the bias in glucose concentrations between CGM and capillary samples were consistent, then the use of a reference food would normalize glucose responses such that CGM could be used to quantify the relative differences in glycemic responses to foods. The data in the current study, however, demonstrate that the magnitude of difference in glucose concentration between CGM and capillary glucose varies by test food, such that the GI for commercially available smoothies was overestimated by CGM to such a degree that it entered the “high GI” category, compared with “medium GI” based on capillary sampling. The reasons for this inaccuracy could be due to the direct effects of the properties of the foods and/or subsequent effects on physiology and metabolism. For example, antioxidants such as vitamin C have been shown to overestimate glucose concentrations with CGM [20], although with 1000 mg vitamin C, the bias in glucose concentration was 0.5 mmol/L, and the vitamin C content of the commercial smoothie was <100 mg. It is, therefore, unlikely that the vitamin C content of the smoothies can explain the inaccuracy of GI quantification. Further support for this comes from the observation that continuous glucose monitoring was able to accurately classify the blended fruit condition (shop-bought fruit in the same recipe as the commercial smoothie and blended into a smoothie), which would also contain vitamin C. The variance between drinks in the accuracy of CGM may, therefore, be more heavily influenced by effects on glucose fluxes between the circulating and interstitial pools. The implications of this are that when accurate quantification of the glycemic response to foods is the primary goal, capillary sampling should be prioritized over continuous glucose monitoring.

The composition and physical structure of food can influence rates of digestion, intestinal absorption, and metabolism of carbohydrates and thus alter glucose kinetics and, potentially, glucose concentrations. We therefore compared the glycemic responses to manipulating the physical form (whole fruit compared with blended), added fiber rate of ingestion, and the dose of carbohydrate. We found no evidence that these factors made a meaningful difference in the glycemic response in either capillary blood or continuous glucose monitoring estimates of glycemia. Prior work has shown that higher doses (25 g) of inulin can substantially lower glycemic responses to orange juice [21]. The current data suggest that adding 5 g inulin was insufficient to meaningfully lower the glycemic response to a fruit smoothie. It is notable that glucose concentrations in response to different meals can be remarkably similar despite substantial differences in glucose kinetics [22]. Therefore, the lack of differences in glucose concentrations does not rule out the possibility that rates of glucose appearance/disappearance were altered by the different types of test foods and ingestion patterns employed, which could have other implications on metabolism. Future work could clarify the dose of inulin required to alter glycemia and changes in other aspects of metabolism, such as lipaemia. Notably, reducing the dose of carbohydrates from PRODUCT to DOSE did not significantly lower the glycemic response. This is consistent with studies showing no significant difference in glycemic responses to ingestion of 25 g, 50 g, and 75 g glucose [23]. This is explained by differences in glucose kinetics (increased blood glucose appearance rates offset by increased blood glucose disappearance rates) [23]. This is also the most likely explanation for a lack of difference in glycemic response between PRODUCT and SLOW. If CGM error is dependent on glucose fluxes, then differences in glucose fluxes between compartments may explain why there was a higher iAUC with CGM compared with the criterion in PRODUCT but not with DOSE.

Differences in glucose fluxes between interstitial and circulating pools may also have implications for the variance in the accuracy of CGM between people. Although claims are often made regarding the interindividual variance of glycemic responses, these claims often overstate the evidence as the interindividual variance is not separated by the individual-by-treatment response variance [[24], [25], [26]]. Understanding interindividual variance requires replication at the level of the individual [24,25,27]. We, therefore, explored the interindividual heterogeneity of the bias in continuous glucose monitoring estimates of fasting glucose using the 7 replicates of fasting glucose concentrations for each of the 15 participants. We found evidence of significant and clinically meaningful interindividual heterogeneity of the interstitial-capillary glucose difference. We subsequently examined whether this could be explained by any basic characteristics of the participants and did not find evidence that the interindividual heterogeneity of the CGM bias of fasting glucose concentrations could be explained by either fasting glucose concentrations, glucose tolerance status, age, or BMI. Since the evening meal prior to trials was standardized within participants but not between participants, then this could also contribute to interindividual variability. Further work is therefore needed to establish the underlying reasons for interindividual heterogeneity of the CGM bias, which may relate to interstitial pool sizes and degrees of fibrotic encapsulation [28]. Another possibility for discordance between criterion and CGM measures could relate to the relatively low reliability of CGMs [11]. Indeed, we report here that there is substantial variability in the bias of fasting glucose concentrations both within-CGMs and between-CGMs. By better understanding these factors, it may be possible to improve the validity of continuous glucose monitoring for assessing glycemia. This may involve some degree of calibration against capillary samples and/or changes in the algorithms. Our data demonstrate that a single calibration of CGM using a fasting capillary glucose sample is not sufficient to fully correct for the bias, and therefore, multiple calibration and/or algorithm adaptation is likely to be required.

Limitations of the current study include the testing of only 1 brand and model of CGM, and therefore, it remains unknown whether other brands and models of CGM display such errors in characterizing glycemic responses. Furthermore, only 1 reference test was performed. GI testing guidelines recommend that the reference test be performed at least twice to improve the precision of the estimate for the glycemic response to the reference food. This is because the reference response becomes the denominator in the calculation of GI for all test foods. Therefore, imprecision in the estimate of the reference can affect the calculation of GI for all of the test foods. This compounding of imprecision is a likely reason for the typical non-normal distribution when 2 normally distributed datasets are expressed as a ratio of one another (such as GI) [29]. Extreme outliers in a non-normal distribution will have a large influence on a mean. Although duplicating reference tests is 1 way to reduce the influence of extreme outliers in non-normally distributed data, another way is to use measures of central tendency that are less influenced by extreme outliers, such as the median or the geometric mean. Interestingly, the GI data in the current study do show some evidence of non-normal distribution, and when the mean is used, the GI of the product is higher than previous estimates using duplicate reference tests (mean ± SD: 60 ± 35 compared with 36 ± 16 [30]). When geometric mean was used, then the estimate of GI was closer to previous estimates (53 ± 2). Whether measures of central tendency such as median or geometric mean can substitute the recommendation for duplicate tests is unclear, and therefore, the estimate of GI from the current study should be considered with caution. The current study also has a relatively small sample of healthy individuals, and thus, generalizability to other populations is unknown.

In conclusion, these data demonstrate that a CGM can overestimate postprandial glycemia to differing degrees across foods/beverages. Consequently, the GI of a fruit smoothie is misclassified using CGM. When using the criterion method of capillary blood, the GI of a typical fruit smoothie is low (with 95% CI spanning low to medium). Interestingly, the glycemic response was not substantially affected by the addition of a modest quantity of fiber, the loss of physical structure of the food, or the dose or speed of ingestion, which may reflect the fact that GI cannot fully capture blood glucose kinetics. When CGM is used to quantify the GI, then differential degrees of error across participants and test drinks can result in overestimation of GI and relative hyperglycemia (time-outside-range). Individual adjustment of the CGM for the difference in fasting glucose concentrations can improve but not eliminate the bias of CGM in postprandial samples. Therefore, if accurate quantification of postprandial glycemia is the primary aim, then capillary blood should be the preferred method, and CGM may not be suitable, at least with respect to the CGM device tested in the present study.

## Author contributions

The authors’ responsibilities were as follows– JTG, KMH, JAB: designed the research; KMH: conducted the research; JTG, KMH, AH, DT, JAB: analyzed the data and performed the statistical analysis and drafted the manuscript; JTG: has primary responsibility for final content; and all authors: read and approved the final manuscript.

## Data availability

Data described in the manuscript, code book, and analytic code will be made publicly and freely available without restriction at https://doi.org/10.17632/3m82byt9s3.1.

## Funding

The funding for the current project was supported by an unrestricted grant from Innocent Drinks. The funder had no involvement in the study design, collection, analysis, or interpretation of data, writing of the manuscript, or publication other than the provision of test products.

## Conflict of interest

For a full list of JTG’s disclosures, see https://gonzalezjt1.wordpress.com/2024/03/**;** JTG has received research funding from BBSRC, MRC, the British Heart Foundation, Clasado Biosciences, Lucozade Ribena Suntory, ARLA Foods Ingredients, and the Cosun Nutrition Center. He is a scientific advisory board member for ZOE and 6d Sports Nutrition and has completed paid consultancy for The Dairy Council, PepsiCo, Violicom Medical, Tour Racing Ltd, the European Fruit Juice Association, and SVGC. JAB is an investigator on research grants funded by BBSRC, MRC, the British Heart Foundation, the Rare Disease Foundation, the EU Hydration Institute, GlaxoSmithKline, Nestlé, Lucozade Ribena Suntory, ARLA Foods, the Cosun Nutrition Center, the American Academy of Sleep Medicine Foundation, Salus Optima (L3M Technologies Ltd), and the Restricted Growth Association. He has completed paid consultancy for PepsiCo, Kellogg’s, SVGC, and Salus Optima (L3M Technologies Ltd). JAB is also the Company Director of Metabolic Solutions Ltd, receives an annual honorarium as a member of the academic advisory board for the International Olympic Committee Diploma in Sports Nutrition, and receives an annual stipend as editor-in-chief of the *International Journal of Sports Nutrition & Exercise Metabolism*. All other authors report no conflicts of interest.

## References

[1] Chen Y.C., Betts J.A., Walhin J.P., Thompson D. (2018). Adipose tissue responses to breaking sitting in men and women with central adiposity. Med. Sci. Sports Exerc..

[2] Merovci A., Tripathy D., Chen X., Valdez I., Abdul-Ghani M., Solis-Herrera C. (2021). Effect of mild physiologic hyperglycemia on insulin secretion, insulin clearance, and insulin sensitivity in healthy glucose-tolerant subjects. Diabetes.

[3] Tripathy D., Merovci A., Basu R., Abdul-Ghani M., DeFronzo R.A. (2019). Mild physiologic hyperglycemia induces hepatic insulin resistance in healthy normal glucose-tolerant participants. J Clin. Endocrinol. Metab..

[4] Gonzalez J.T., Stevenson E.J. (2012). Postprandial glycemia and appetite sensations in response to porridge made with rolled and pinhead oats. J Am. Coll. Nutr..

[5] Brouns F., Bjorck I., Frayn K.N., Gibbs A.L., Lang V., Slama G. (2005). Glycaemic index methodology. Nutr. Res. Rev..

[6] Edinburgh R.M., Hengist A., Smith H.A., Betts J.A., Thompson D., Walhin J.P. (2017). Prior exercise alters the difference between arterialised and venous glycaemia: implications for blood sampling procedures. Br. J Nutr..

[7] Berry S.E., Valdes A.M., Drew D.A., Asnicar F., Mazidi M., Wolf J. (2020). Human postprandial responses to food and potential for precision nutrition. Nat. Med..

[8] Guess N. (2023). The growing use of continuous glucose monitors in people without diabetes: an evidence-free zone, Pract. Diabetes.

[9] FDA U.S. Food and Drug Administration Publisher FDA. https://www.fda.gov/news-events/press-announcements/fda-clears-first-over-counter-continuous-glucose-monitor.

[10] Bailey T., Bode B.W., Christiansen M.P., Klaff L.J., Alva S. (2015). The performance and usability of a factory-calibrated flash glucose monitoring system, Diabetes Technol. Ther.

[11] Hengist A., Ong J.A., McNeel K., Guo J., Hall K.D. (2025). Imprecision nutrition? Intraindividual variability of glucose responses to duplicate presented meals in adults without diabetes. Am. J Clin. Nutr..

[12] Howard R., Guo J., Hall K.D. (2020). Imprecision nutrition? Different simultaneous continuous glucose monitors provide discordant meal rankings for incremental postprandial glucose in subjects without diabetes. Am. J Clin. Nutr..

[13] WMA (2013).

[14] Atkinson F.S., Brand-Miller J.C., Foster-Powell K., Buyken A.E., Goletzke J. (2021). International tables of glycemic index and glycemic load values 2021: a systematic review. Am. J Clin. Nutr..

[15] Narang B.J., Atkinson G., Gonzalez J.T., Betts J.A. (2020). A tool to explore discrete-time data: the time series response analyser. Int. J Sport Nutr. Exerc. Metab..

[16] Battelino T., Danne T., Bergenstal R.M., Amiel S.A., Beck R., Biester T. (2019). Clinical targets for continuous glucose monitoring data interpretation: recommendations from the international consensus on time in range. Diabetes Care.

[17] Cohen J. (1988).

[18] Cook J.A., Julious S.A., Sones W., Hampson L.V., Hewitt C., Berlin J.A. (2018). DELTA2 guidance on choosing the target difference and undertaking and reporting the sample size calculation for a randomised controlled trial. BMJ.

[19] Rao Kondapally Seshasai S., Kaptoge S., Thompson A., Di Angelantonio E., Gao P., Sarwar N. (2011). Diabetes mellitus, fasting glucose, and risk of cause-specific death, N Engl. J Med.

[20] Heinemann L. (2022). Interferences with CGM systems: practical relevance?. J Diabetes Sci. Technol..

[21] Steinert R.E., Mueller M., Serra M., Lehner-Sigrist S., Frost G., Gero D. (2024). Effect of inulin on breath hydrogen, postprandial glycemia, gut hormone release, and appetite perception in RYGB patients: a prospective, randomized, cross-over pilot study. Nutr. Diabetes.

[22] Eelderink C., Schepers M., Preston T., Vonk R.J., Oudhuis L., Priebe M.G. (2012). Slowly and rapidly digestible starchy foods can elicit a similar glycemic response because of differential tissue glucose uptake in healthy men. Am. J Clin. Nutr..

[23] Kowalski G.M., Moore S.M., Hamley S., Selathurai A., Bruce C.R. (2017). The effect of ingested glucose dose on the suppression of endogenous glucose production in humans. Diabetes.

[24] Senn S. (2016). Mastering variation: variance components and personalised medicine. Stat. Med..

[25] Senn S. (2018). Statistical pitfalls of personalized medicine. Nature.

[26] Atkinson G., Batterham A.M. (2015). True and false interindividual differences in the physiological response to an intervention. Exp. Physiol..

[27] Gonzalez J.T., Lolli L., Veasey R.C., Rumbold P.L., Betts J.A., Atkinson G. (2024). Are there interindividual differences in the reactive hypoglycaemia response to breakfast? A replicate crossover trial. Eur. J Nutr.

[28] McClatchey P.M., McClain E.S., Williams I.M., Malabanan C.M., James F.D., Lord P.C. (2019). Fibrotic encapsulation is the dominant source of continuous glucose monitor delays. Diabetes.

[29] Geary R.C. (1930). The frequency distribution of the quotient of two normal variates. J R Stat. Soc..

[30] Saltaouras G., Shaw P.K., Fraser A.C., Hawes C., Smith H., Handley L. (2019). Glycaemic index, glycaemic load and dietary fibre characteristics of two commercially available fruit smoothies. Int. J Food Sci. Nutr..

