# Supplementary material for: Continuous glucose monitor overestimates glycemia, with the magnitude of bias varying by postprandial test and individual – a randomized crossover trial
Source: Am J Clin Nutr. 2025 Feb 26;121(5):1025–34. doi: 10.1016/j.ajcnut.2025.02.024 (PMC12107490; doi:10.1016/j.ajcnut.2025.02.024)
Supplement: Multimedia component 1 [file mmc1.pdf]

Continuous glucose monitor overestimates glycemia, with the magnitude of bias varying by postprandial test and individual – A randomized crossover trial.

Hutchins et al.

Supplementary Material

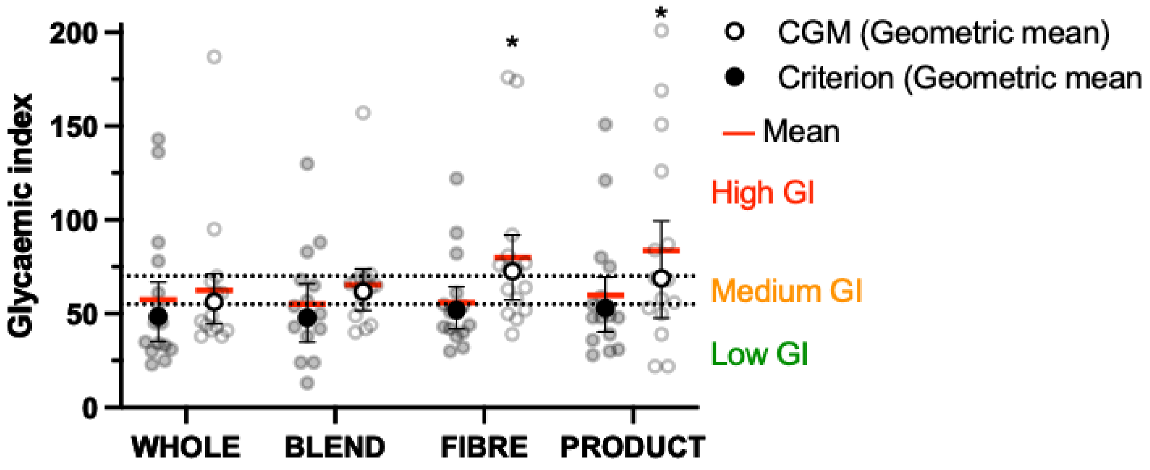

Supplementary Figure 1. Glycemic index determined using continuous glucose monitor (CGM) or capillary blood (criterion) in response to ingestion of 50 g of carbohydrate as whole fruit (WHOLE), blended fruit (BLEND), commercially available fruit smoothie ingested within 5 minutes (PRODUCT) or ingested with 5 g inulin (FIBRE). *n* = 15 healthy adults. Individual data are shown alongside the mean and geometric mean ± 95%CI. **p* < 0.05 for CGM *versus* criterion.

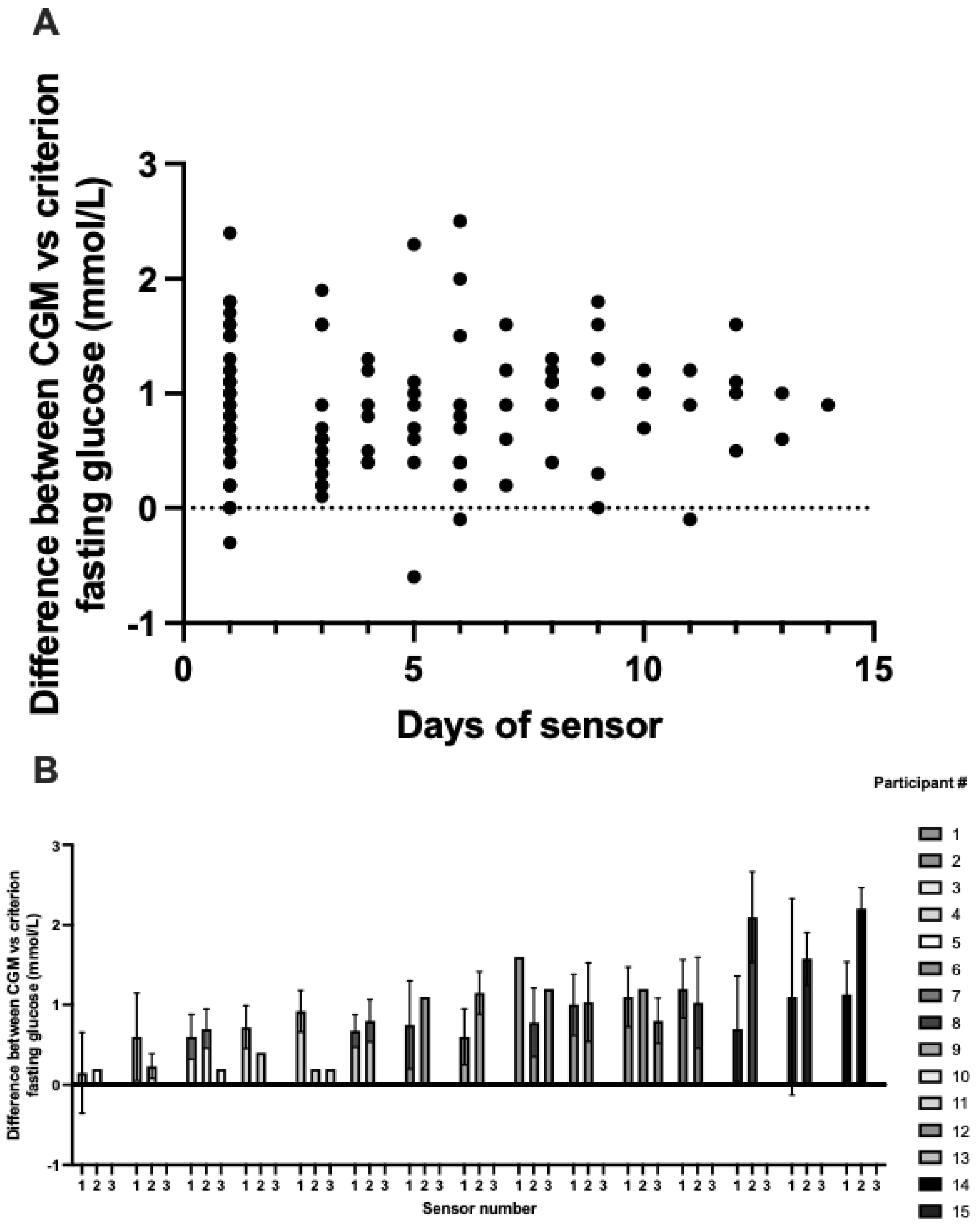

Supplementary Figure 2. (A) Difference in fasting continuous glucose monitor (CGM) *versus* criterion (capillary blood) glucose concentration in relation to the days of sensor wear. **(B)** Mean ± SD for the difference in fasting continuous glucose monitor (CGM) *versus* criterion (capillary blood) glucose concentration for each participant broken down by sensor number.

Supplementary Table 1. Conditions for each participant across each laboratory visit.

	Participant 1	Participant 2	Participant 3	Participant 4	Participant 5	Participant 6	Participant 7	Participant 8	Participant 9	Participant 10	Participant 11	Participant 12	Participant 13	Participant 14	Participant 15
**Visit 1**	WHOLE	PRODUCT	CONTROL	SLOW	PRODUCT	SLOW	CONTROL	WHOLE	PRODUCT	CONTROL	BLEND	PRODUCT	SLOW	BLEND	FIBRE
**Visit 2**	DOSE	SLOW	FIBRE	BLEND	FIBRE	CONTROL	BLEND	SLOW	BLEND	FIBRE	DOSE	CONTROL	CONTROL	FIBRE	WHOLE
**Visit 3**	SLOW	DOSE	SLOW	DOSE	WHOLE	FIBRE	SLOW	PRODUCT	SLOW	PRODUCT	CONTROL	WHOLE	BLEND	PRODUCT	DOSE
**Visit 4**	CONTROL	FIBRE	BLEND	FIBRE	CONTROL	WHOLE	DOSE	FIBRE	DOSE	BLEND	PRODUCT	SLOW	DOSE	SLOW	CONTROL
**Visit 5**	BLEND	BLEND	PRODUCT	CONTROL	DOSE	BLEND	WHOLE	BLEND	FIBRE	WHOLE	FIBRE	BLEND	FIBRE	WHOLE	PRODUCT
**Visit 6**	PRODUCT	WHOLE	DOSE	PRODUCT	SLOW	PRODUCT	PRODUCT	DOSE	CONTROL	DOSE	SLOW	DOSE	WHOLE	CONTROL	SLOW
**Visit 7**	FIBRE	CONTROL	WHOLE	WHOLE	BLEND	DOSE	FIBRE	CONTROL	WHOLE	SLOW	WHOLE	FIBRE	PRODUCT	DOSE	BLEND

Whole fruit (WHOLE), blended fruit (BLEND), commercially available fruit smoothie ingested within 5 minutes (PRODUCT), as a 30 g dose of carbohydrate (DOSE), ingested over ~30 minutes (SLOW), ingested with 5 g inulin (FIBRE), and 50 g glucose (CONTROL).
